# Coexistence of Acute Myeloid Leukemia and B-cell Non-Hodgkin Lymphoma Diagnosed on a Bone Marrow Trephine Biopsy

**DOI:** 10.7759/cureus.81107

**Published:** 2025-03-24

**Authors:** Natalia Ahmad, Hiba Asif, Maryam Burhan, Asad H Ahmad, Muhammad Tariq Mahmood

**Affiliations:** 1 Pathology and Laboratory Medicine, Shaukat Khanum Memorial Cancer Hospital and Research Centre, Lahore, PAK

**Keywords:** acute myeloid leukemia (aml), b-cell non-hodgkin lymphoma (b-nhl), bone marrow trephine biopsy, immunohistochemical stains (ihc), low-grade b-cell non-hodgkin lymphoma

## Abstract

Acute myeloid leukemia (AML) and low-grade B-cell non-Hodgkin lymphoma (B-NHL), both malignant hematological cancers, are primarily different diseases. The coexistence of these two malignancies at initial diagnosis is rare and often leads to unique challenges in the treatment of the patient. Here, we present a case of a 70-year-old male who presented with pancytopenia on peripheral blood and was diagnosed with concurrent AML and low-grade B-NHL on a bone marrow trephine biopsy. He was diagnosed solely on a bone marrow trephine biopsy as his aspirate was diluted and inadequate for flow cytometry. There are only a small number of case reports with the coexistence of AML and low-grade B-NHL.

## Introduction

The clonal growth of myeloid blasts in the bone marrow, bloodstream, and other tissues is a characteristic of acute myeloid leukemia (AML), a diverse group of malignant tumors. AML is the most common type of acute leukemia found in adults, with a typical diagnosis occurring at a median age of 65. This condition shows a slight predominance in males across various countries and is linked to a poor prognosis [1,2]. Low-grade B-cell non-Hodgkin lymphoma (B-NHL) is a form of cancer that arises from an abnormal clone of B lymphocytes. It is marked by the gradual proliferation of cancer cells that can build up in the lymph nodes, spleen, bone marrow, and various lymphatic tissues. The occurrence of low-grade B-NHL rises with age, with the median diagnosis age being 60-70 years [1]. Non-Hodgkin lymphoma (NHL) is an important group of malignancies that are highly treatable and potentially curable [3]. AML and B-NHL are two distinct hematological malignancies that typically arise from different cellular lineages, with AML originating from the myeloid stem cells and B-NHL originating from B lymphocytes. The simultaneous occurrence of these two malignancies, known as "double malignancy," is a rare clinical event. The coexistence of AML and B-NHL at initial diagnosis is an exceptional and complex scenario as it presents both diagnostic and therapeutic challenges [4]. This case report will discuss the rarity, pathophysiological mechanisms, clinical presentation, and strategies associated with the simultaneous diagnosis of AML and B-NHL.

## Case presentation

**Figure 1:**
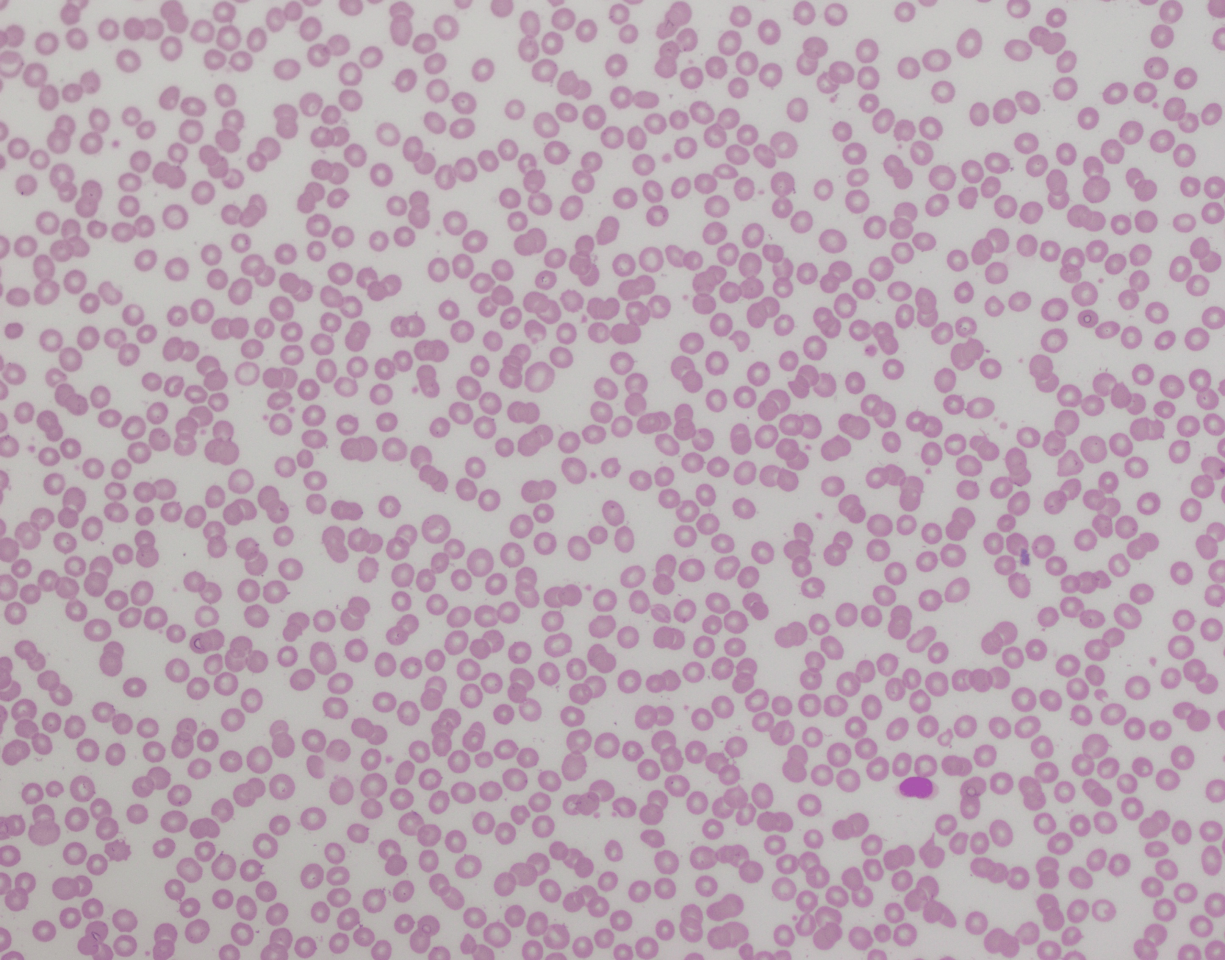
Peripheral blood showing pancytopenia

**Figure 2:**
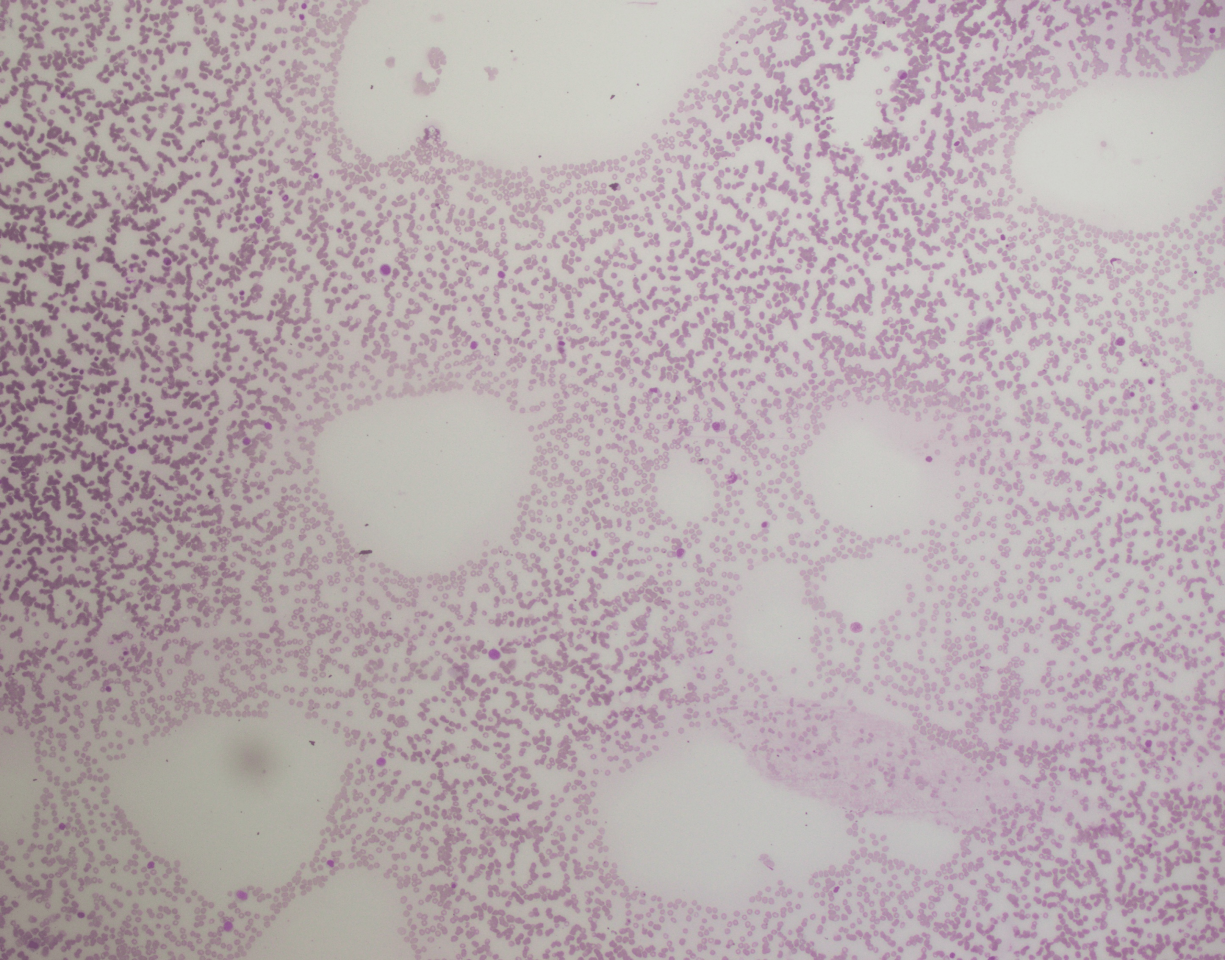
Bone marrow aspirate (hemodiluted)

**Figure 3:**
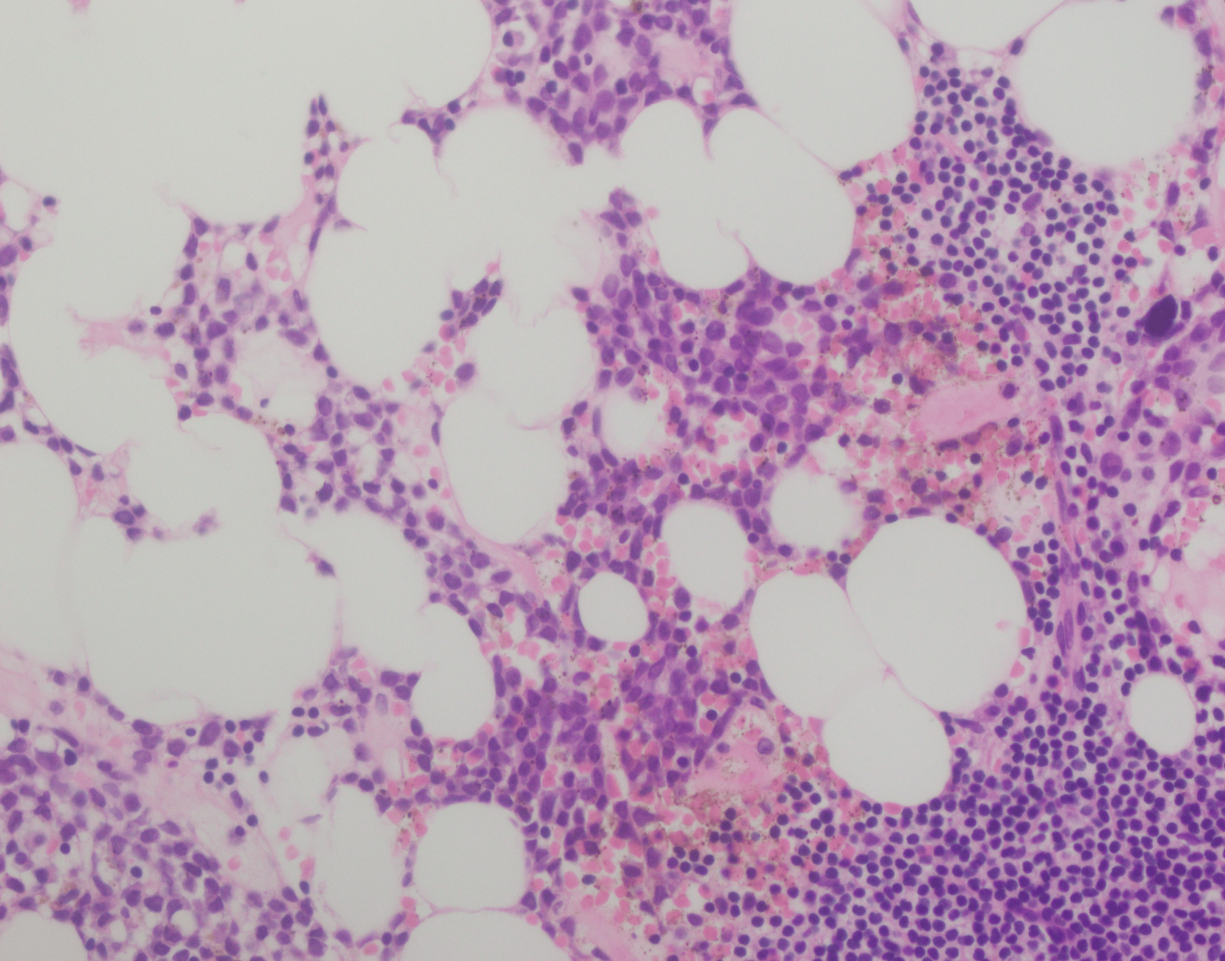
Trephine biopsy showing interstitial mononuclear cells with open chromatin

**Figure 4:**
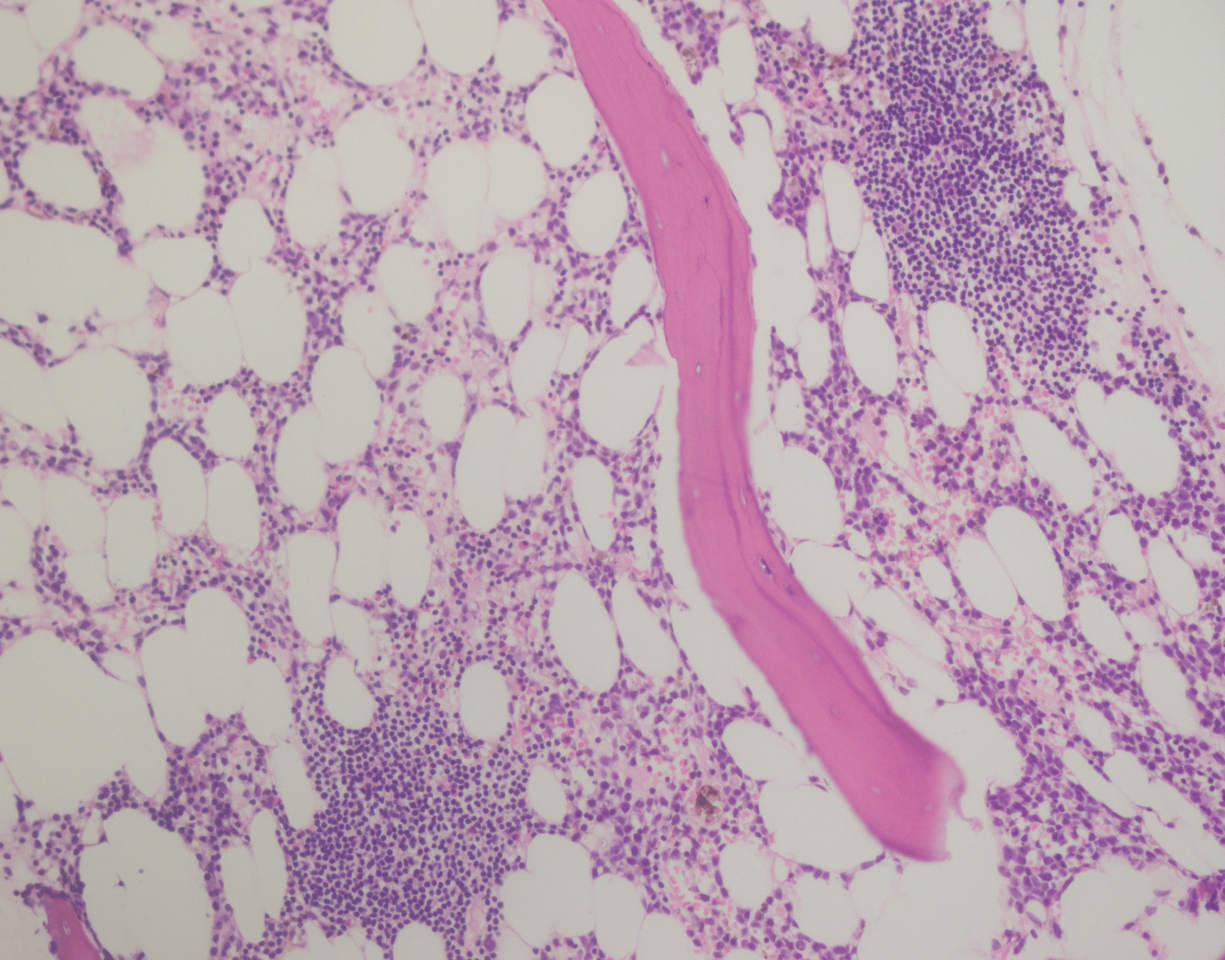
Trephine biopsy showing multiple lymphoid aggregates

**Figure 5:**
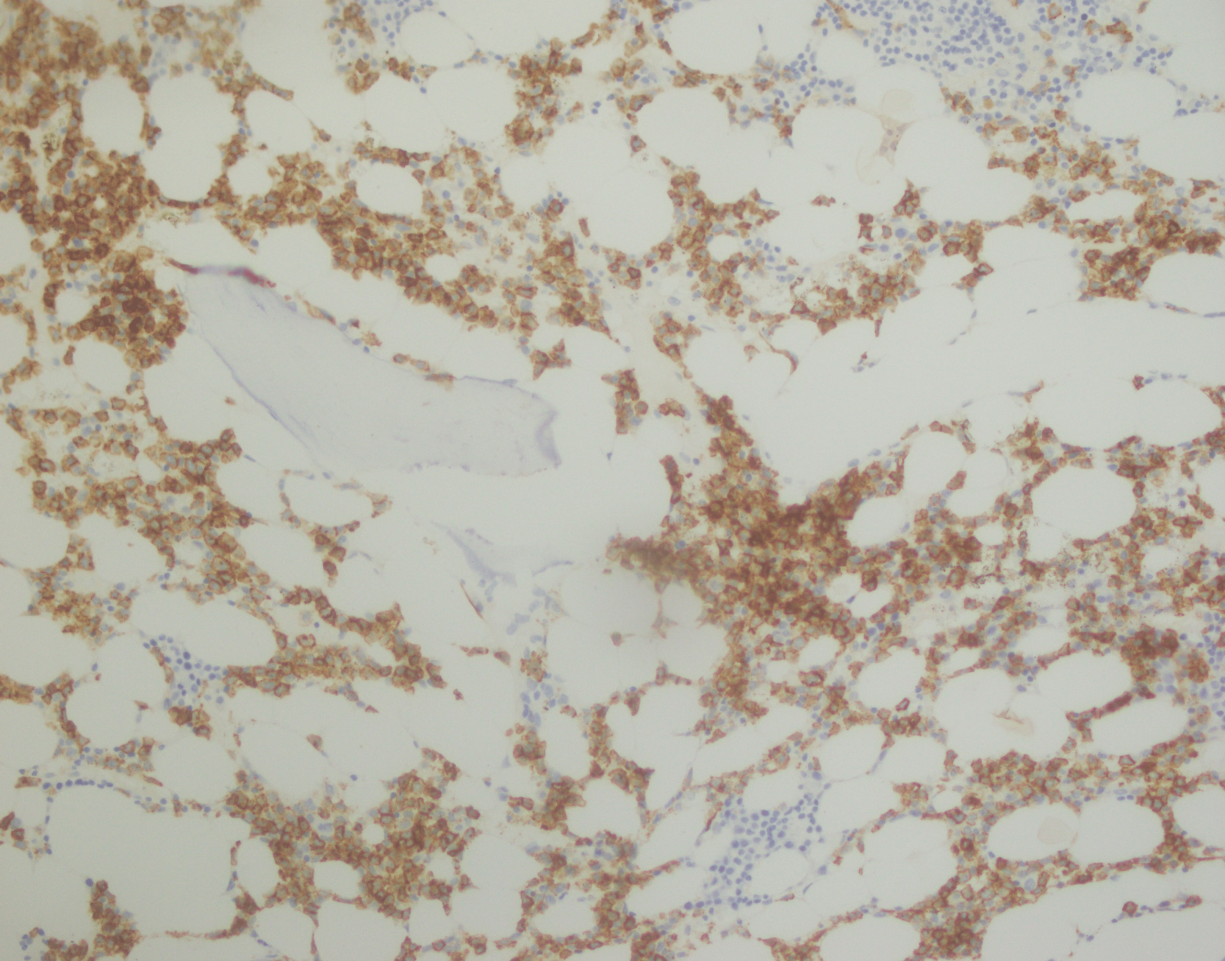
CD34 immunostaining positive in blasts

**Figure 6:**
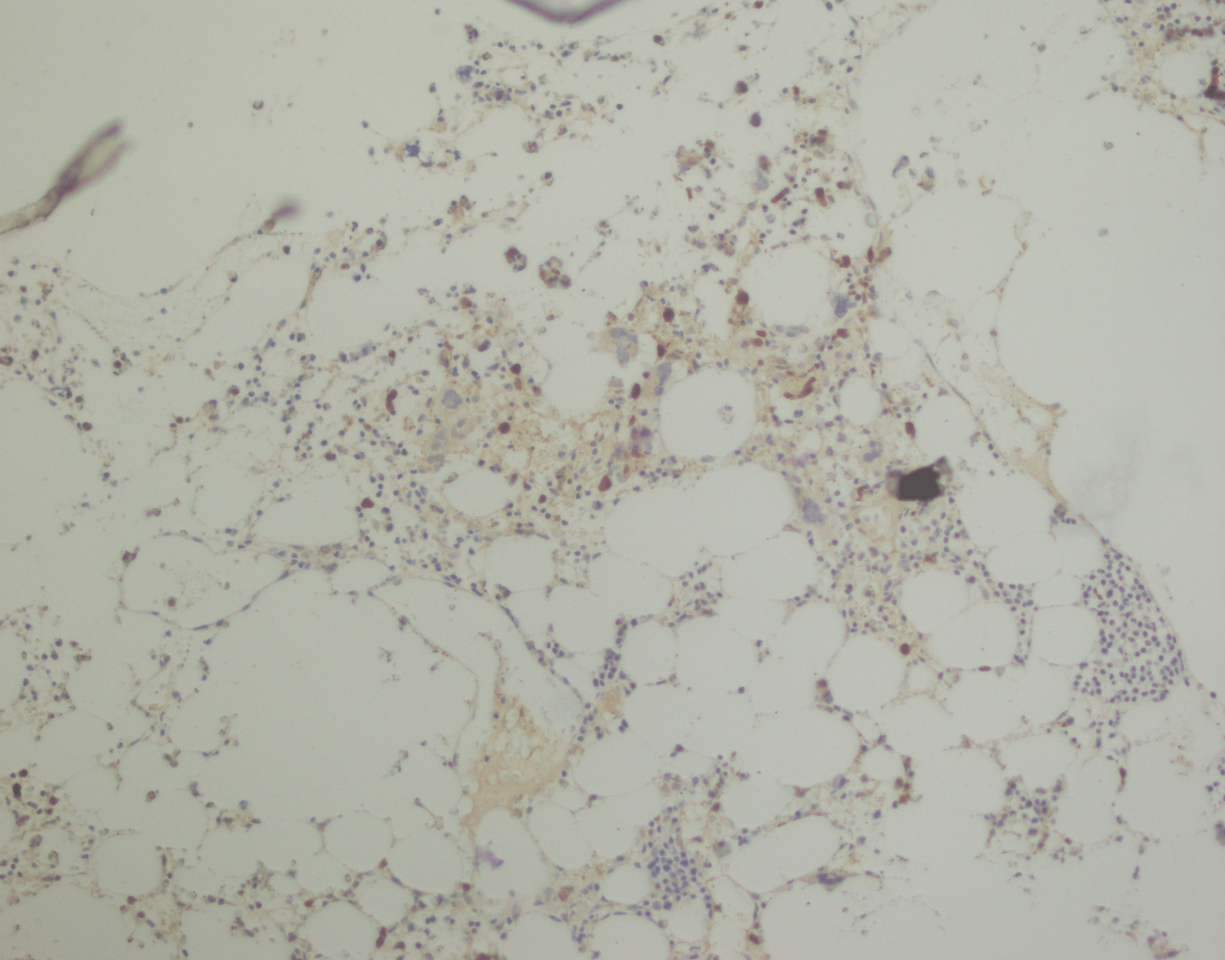
TDT (terminal deoxynucleotidyl transferase) immunostaining positive in blasts

**Figure 7:**
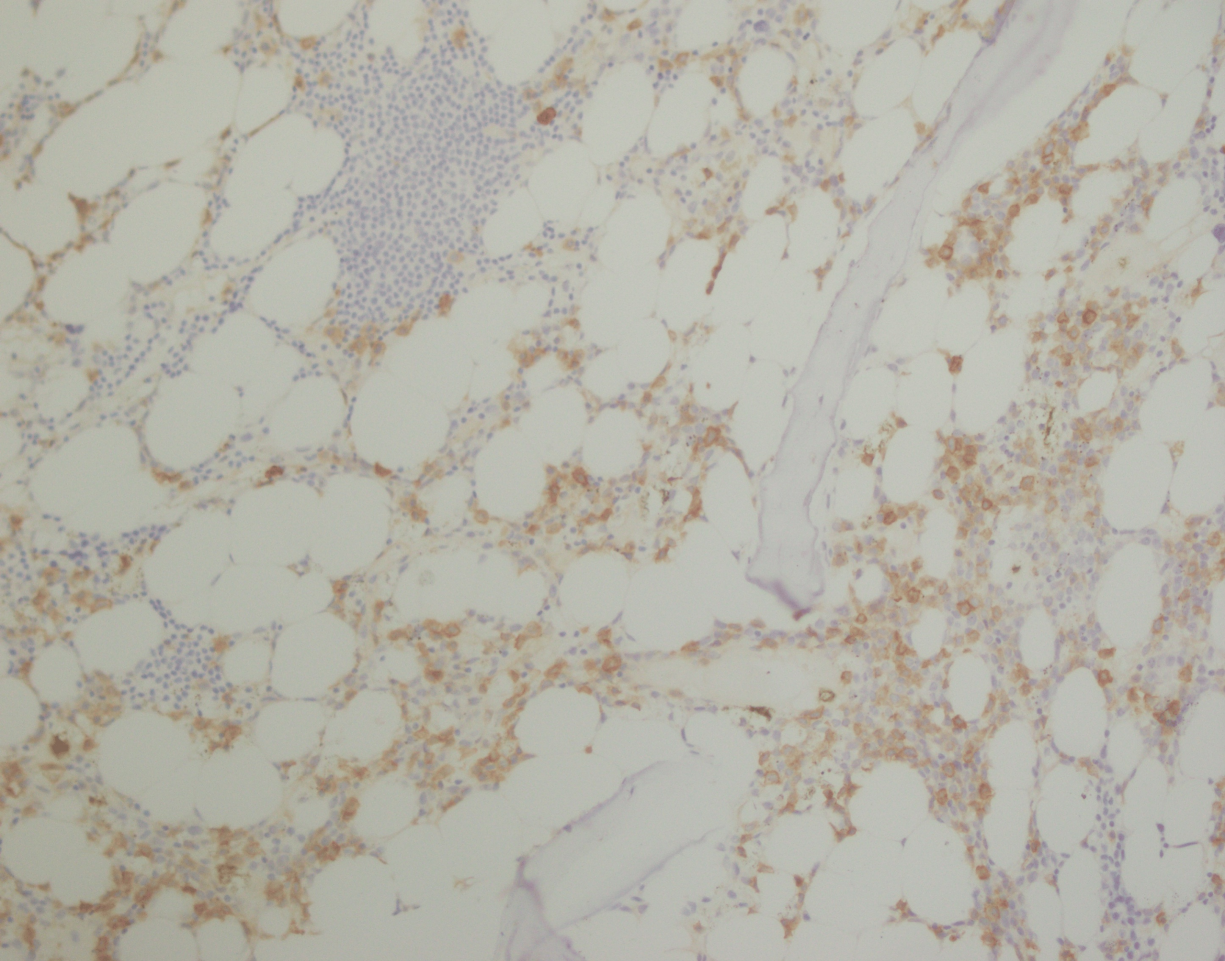
CD117 immunostaining positive in blasts

**Figure 8:**
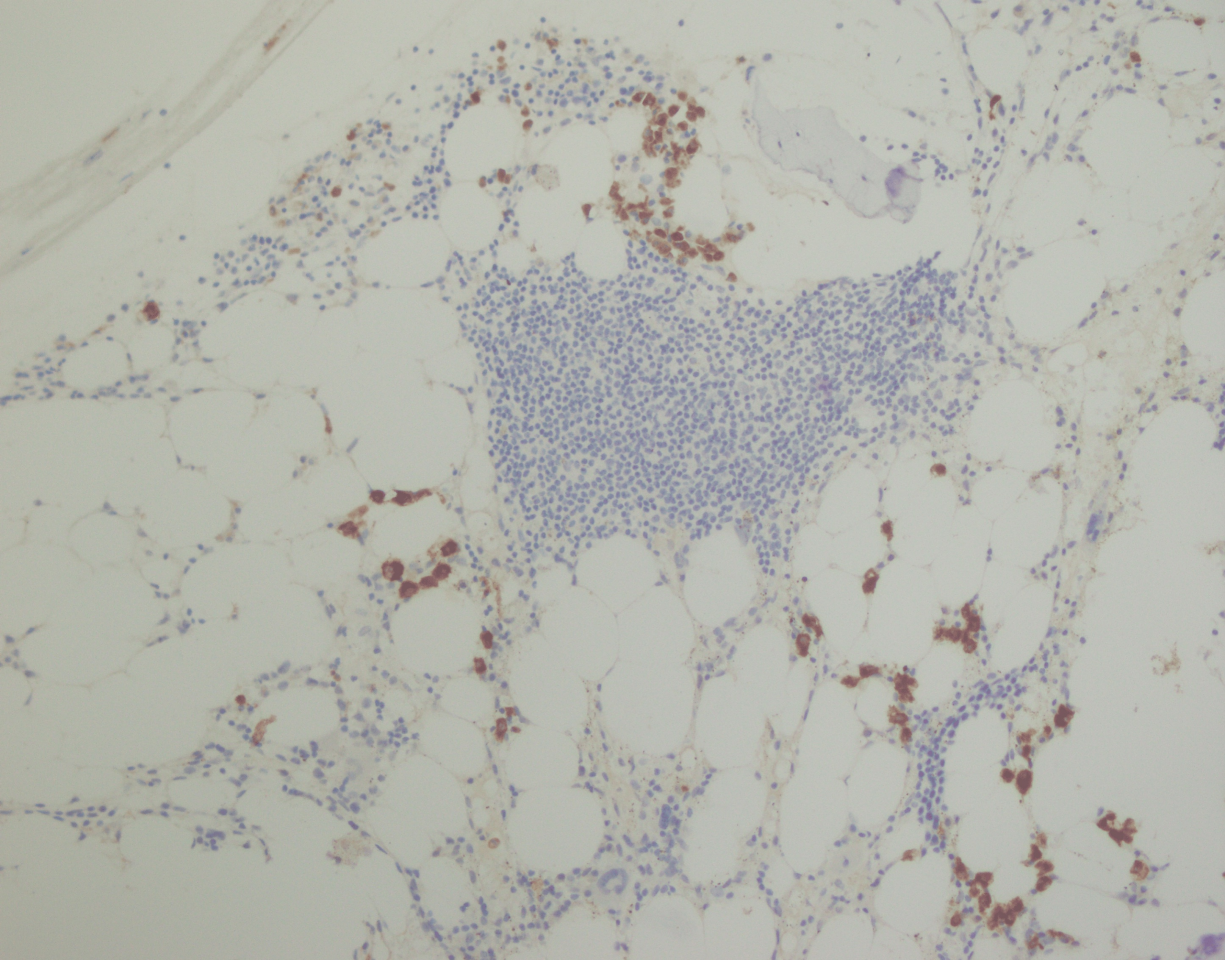
MPO (myeloperoxidase) immunostaining positive in blasts

**Figure 9:**
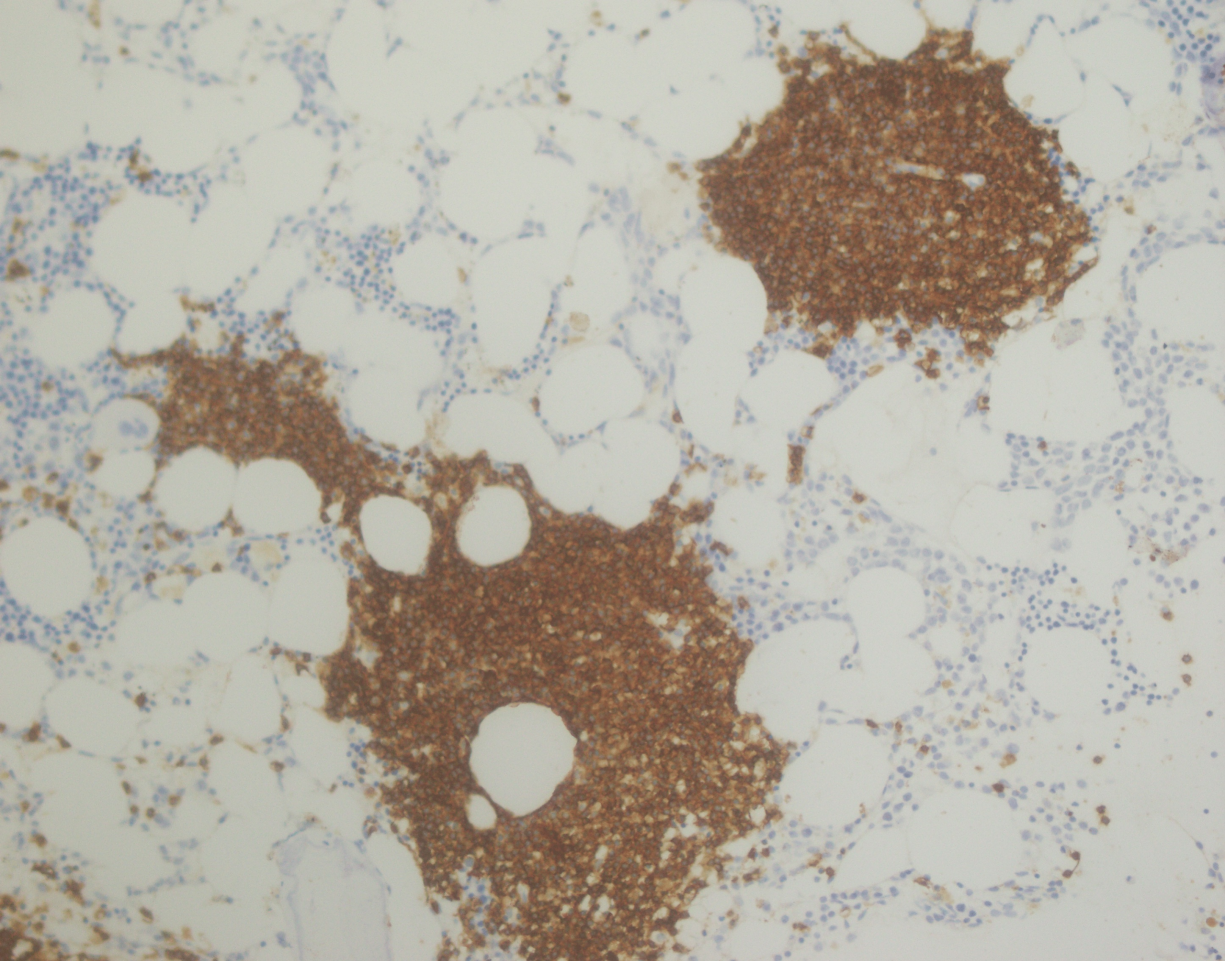
CD20 immunostaining positive in lymphoid aggregates

**Figure 10:**
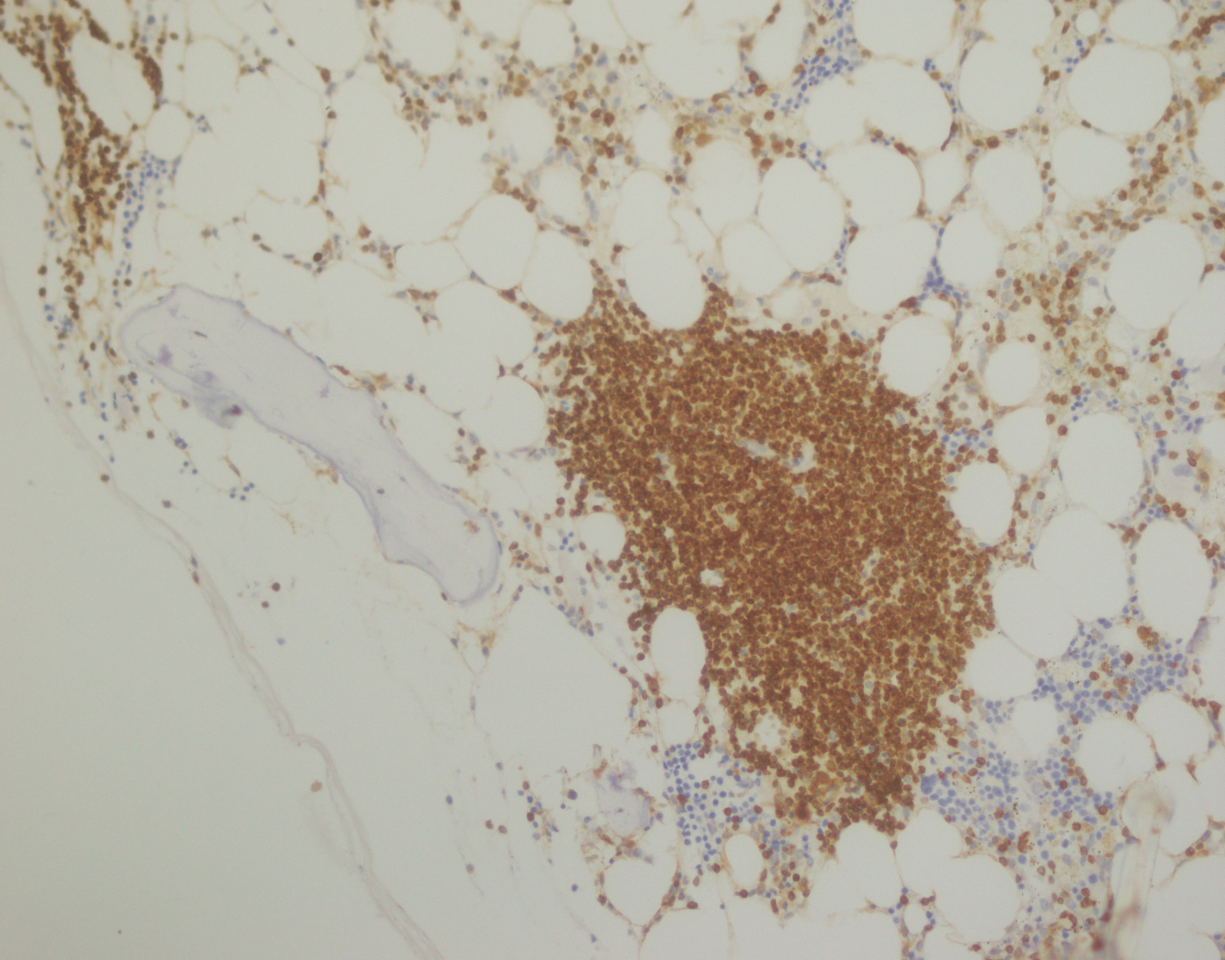
BCL-2 immunostaining positive in lymphoid aggregates

**Figure 11:**
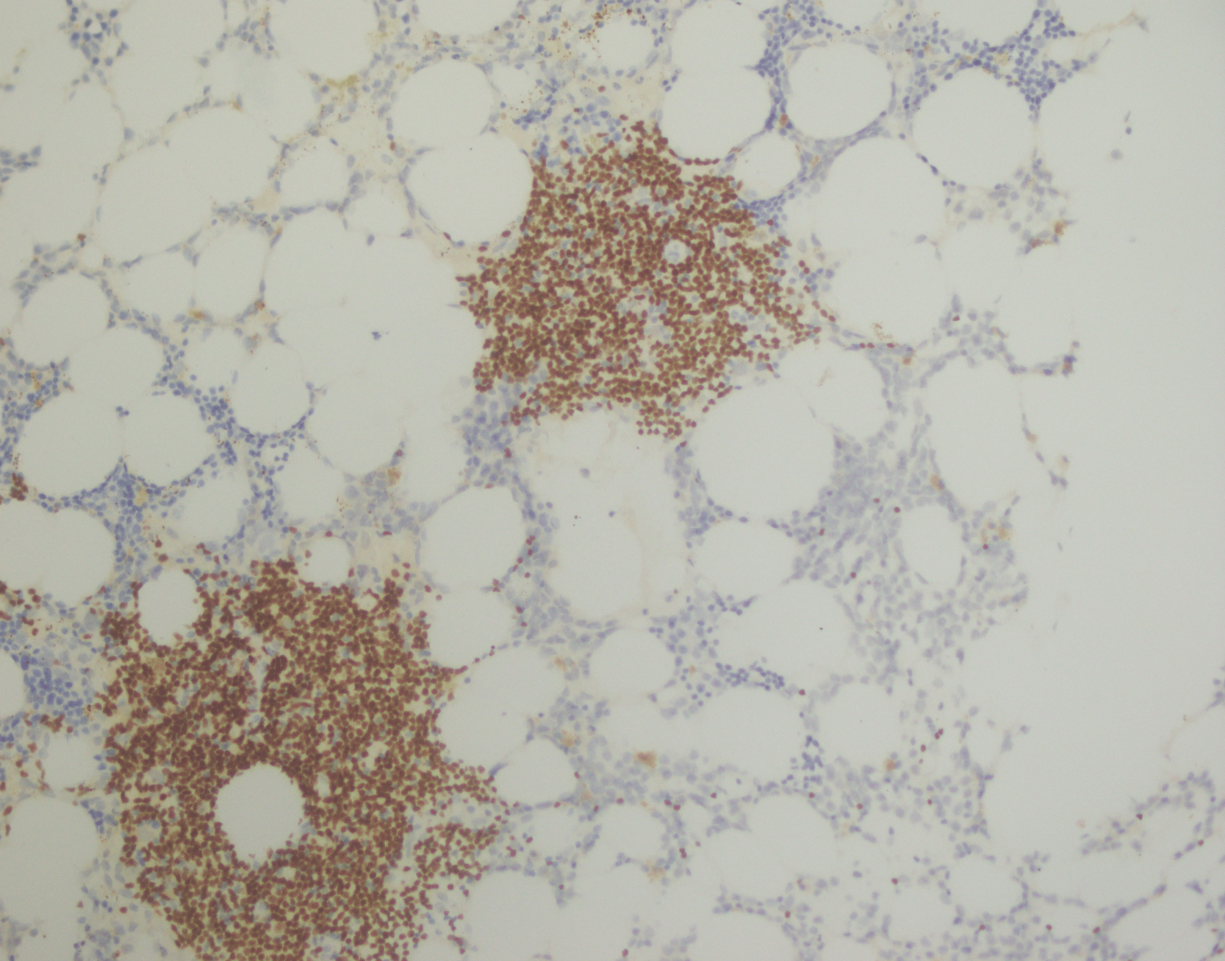
PAX5 immunostaining positive in lymphoid aggregates

**Figure 12:**
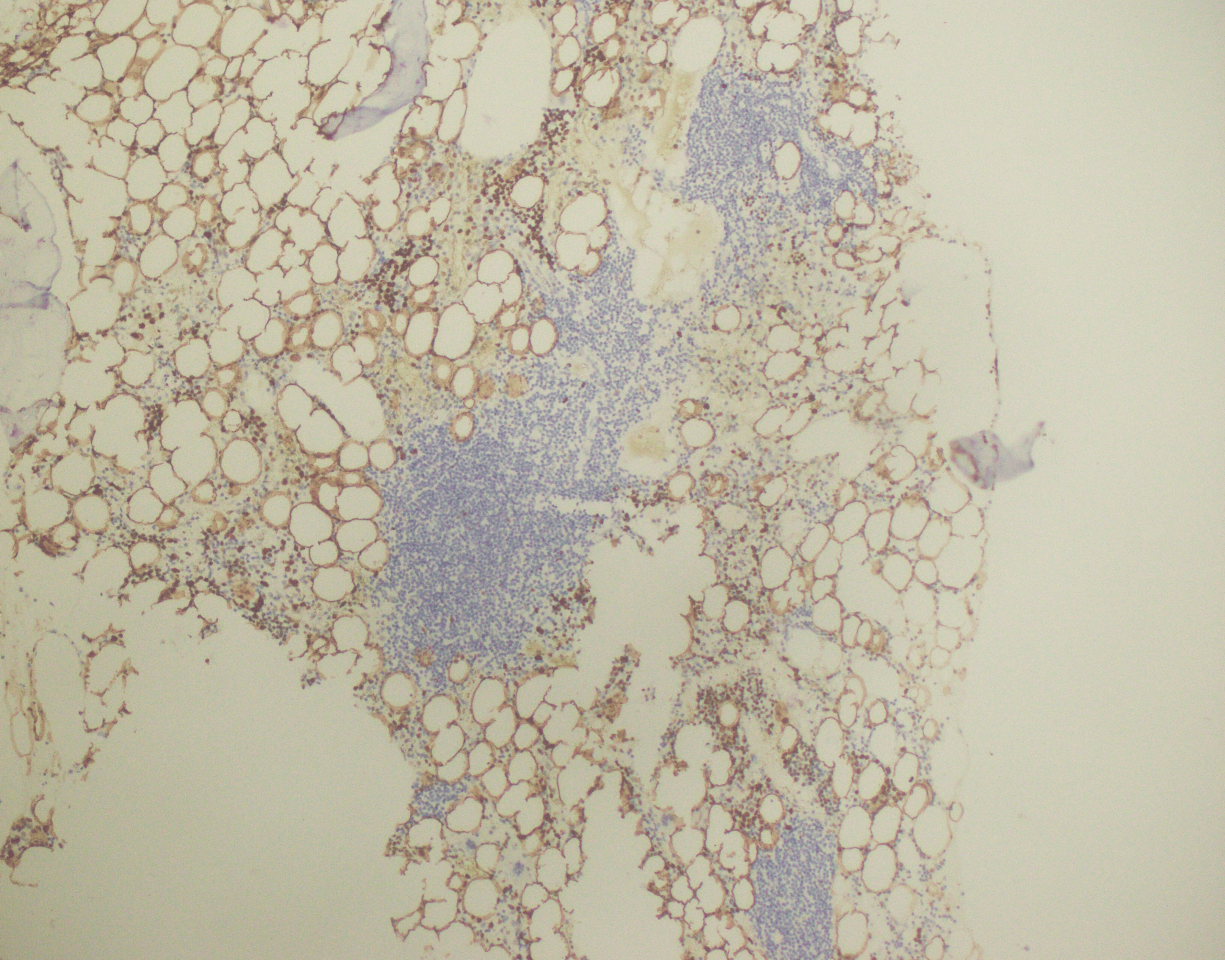
Ki-67 immunostaining showing a low proliferation index

A 70-year-old male presented with fever and sore throat for three weeks. Complete blood count (CBC) revealed pancytopenia with hemoglobin (Hb) of 9.2 g/dl, total leukocyte count (TLC) of 0.88×10^3^/ul, and platelets of 91×10^3^ (Table 1). The patient was advised of a bone marrow biopsy with a suspicion of leukemia. Peripheral blood showed pancytopenia (Figure 1) with no blast cells. Bone marrow aspirate was hemodiluted (Figure 2), and no blast cells were seen. Flow cytometry and cytogenetics were not done, as the bone marrow aspirate was diluted and inadequate. Bone marrow trephine revealed interstitial infiltrate of medium-sized mononuclear cells with open chromatin (Figure 3); however, there was an incidental finding of multiple lymphoid cell aggregates (Figure 4) having clumped chromatin throughout the trephine biopsy. In order to confirm whether these lymphoid aggregates were reactive or clonal, a panel of immunohistochemical stains was performed. The interstitial mononuclear cells were positive for CD34 (Figure 5), TDT (terminal deoxynucleotidyl transferase) (Figure 6), CD117 (Figure 7), and MPO (myeloperoxidase) (Figure 8), favoring AML, while the lymphoid aggregates were positive for CD20 (Figure 9), Bcl-2 (Figure 10), and PAX5 (Figure 11) and negative for CD5, CD10, CD23, and cyclin D1. This raised the differential diagnosis of CD5 and CD10-negative lymphomas, which include lymphoplasmacytic lymphoma, splenic marginal zone lymphoma, and hairy cell leukemia. Morphology on trephine biopsy was not favoring hairy cell leukemia, and there was no rouleaux formation on peripheral blood or increased plasma cells and lymphoplasmacytoid cells on trephine biopsy; therefore, both entities were ruled out. The third differential diagnosis was splenic marginal zone lymphoma. Keeping in view that splenic marginal zone lymphoma is a low-grade lymphoma, Ki-67 was ordered. The low proliferation index on Ki-67 (Figure 12) favored the presence of low-grade B-NHL (splenic marginal zone lymphoma). It was an unusual finding of dual pathology in bone marrow at initial diagnosis, which was diagnosed exclusively on a bone marrow trephine biopsy due to the unavailability of flow cytometry and cytogenetics. Fluorescence in situ hybridization (FISH) for BCR-ABL and polymerase chain reaction (PCR) for FLT3-ITD, D835 were negative. The patient was explained about treatment options and the prognosis of the disease.

**Table 1:** Complete blood count parameters with reference ranges

Complete blood count parameters	Patient values	Reference ranges
Hemoglobin (Hb) g/dl	9.2	13-16
Total leukocyte count (TLC) (x10^3^/ul)	0.88	4.52-10.93
Platelets (x10^3^/ul)	91	150-450

## Discussion

Myeloid leukemia and lymphoma are different malignancies originating from two lineages and possess separate cytogenetic, cell phenotype, and biological processes [4]. While the majority of cases describe one malignancy preceding or following the other, their coexistence at the outset presents significant diagnostic and therapeutic challenges. Commonly, patients with a prior history of chemotherapy or radiation therapy for another malignancy may develop secondary leukemias [5]. The pathophysiology of the occurrence of two or more simultaneous hematologic malignancies is not well understood, and there have been multiple proposed theories. The simultaneous presence of these two malignancies at initial diagnosis is exceedingly rare; only a small number of case reports have been reported in the literature.

Ke et al. [6] published a case report of a 57-year-old man who presented with complaints of lethargy and swellings on the neck. Examination revealed a number of enlarged superficial lymph nodes all over the body. His CBC revealed low hemoglobin (Hb) and platelet count but normal counts of white blood cells. Cytology of two cervical lymph nodes revealed the presence of NHL. On PET/CT, there were multiple enlarged lymph nodes with hypermetabolism, along with diffuse hypermetabolism of the bone marrow, raising the suspicion of lymphoma infiltration in the bone marrow, but a bone marrow biopsy revealed AML. Eventually, the patient was diagnosed with NHL and AML. Another case reported by Bhar et al. [7] of dual hematological malignancy in an 85‐year‐old male in which the peripheral blood and bone marrow examination revealed two populations of atypical cells, one population comprising large cells with fine chromatin having nuclear folding and the other one consisted of small mature‐appearing lymphocytes. Flow cytometric immunophenotyping confirmed one population of cells to be of monocytic lineage, whereas the other population showed the immunophenotype consistent with chronic lymphocytic leukemia (CLL). Similarly, Licci et al. [8] reported a case of concurrent CLL and AML in an 86-year-old untreated patient, with a detailed immunohistochemical study to confirm the presence of the two different diseases in the same pathological tissue.

Our case of a 70-year-old male with fever and pancytopenia on peripheral blood raised suspicion of leukemia, but lymphoma was less likely due to the absence of any palpable lymph nodes. Bone marrow biopsy, on the other hand, revealed mononuclear cells positive for CD34, TDT, CD117, and MPO, confirming AML along with an incidental finding of multiple lymphoid aggregates staining CD20, PAX5, and Bcl-2, raising a differential diagnosis of low-grade B-NHL, confirmed on Ki-67 stain. Confirmation of dual pathology in bone marrow leads to further molecular testing, including an AML panel. The AML panel also came out to be negative. This case became a therapeutic challenge because of the coexistence of two malignancies in old age. The patient was explained about treatment options and prognosis.

In this specific case, the presence of multiple lymphoid aggregates staining CD20 suggested a concomitant hematopoietic disease, otherwise without immunohistochemical stains it could be overlooked. It is advisable to examine complete bone marrow including aspirate and trephine biopsy and carry out an immunohistochemical stain panel providing more precise information on all cellular components of the tissue in order to prevent missing any findings other than suspected pathology.

## Conclusions

The coexistence of AML and B-NHL in our patient underscores the intricate interplay between immune dysregulation and oncogenesis. The rarity of such a dual diagnosis necessitates a comprehensive diagnostic workup, including histopathological, immunohistochemical, and molecular studies. In the case of diluted bone marrow aspirate, trephine biopsy and immunohistochemical stains are key to the diagnosis of a dual pathology.
